# Supplementary material for: Strong-field ionization of clusters using two-cycle pulses at 1.8 μm
Source: Sci Rep. 2016 Dec 23;6:39664. doi: 10.1038/srep39664 (PMC5180105; doi:10.1038/srep39664)
Supplement: Supplementary Information [file srep39664-s1.pdf]

Strong-field ionization of clusters using two-cycle pulses at 1.8 μm - Supplementary Information

Bernd Schütte[1], Peng Ye[1], Serguei Patchkovskii[2], Dane R. Austin[1], Christian Brahms[1], Christian Strüber[1], Tobias Witting[1], Misha Yu. Ivanov[1,2], John W. G. Tisch[1], and Jon P. Marangos[1]

[1]Department of Physics, Imperial College London, South Kensington Campus, London SW7 2AZ, UK.
[2]Max-Born-Institut, Max-Born-Strasse 2A, 12489 Berlin, Germany.

ABSTRACT

This Supplementary Information provides details about the TDSE calculation in Ar atoms.

The photoelectron spectrum was calculated using the infinite-time surface-flux method with Volkov functions[1] on a spherical product grid of final photoelectron momenta. The photoelectron momentum magnitude was sampled with 3600 points, uniformly distributed on the [0:18] [Bohr/jiffy] interval (corresponding to the maximum of the kinetic energy of about 4.4 keV). Due to the axial symmetry of the dipole Hamiltonian, the angular grid consisted of 361 points, uniformly distributed on the [0:pi] interval for the polar angle θ. The time-dependent flux was sampled on a sphere 137 Bohr away from the origin, sufficient to eliminate the residual effects of the long-range potential for both the direct and rescattered electrons.[1]

We verified that our calculation is converged with respect to the time step, the radial grid density, and the photoelectron momentum grid. Several potential sources of inaccuracies remain in the calculations, namely:

1. In order to improve the numerical efficiency of the calculation, the effective potential[1] adds a large repulsive potential in the inner-core region, which is expected to be sampled by the back-scattered recollision electrons. We expect this modification to somewhat *increase* the probability of a hard recollision event. Therefore, our calculation is expected to *overestimate* the yield of high-energy recollision electrons. Although more accurate effective potentials are available (e.g.[2]), they lead to a very stiff TDSE, which makes time propagation unaffordable for an intense field at 1.8 μm.

2. Our angular grid is fully converged for the direct electrons, but lacks the very high angular momenta (up to $L = 2500$) needed to describe the fine interference fringes formed by the 10 Up electrons. Using such large grids is possible, but increases the cost of TDSE solution by a factor of $\approx$25 (large L increase TDSE stiffness, making smaller time steps necessary). From our previous experience, the total yields at a given photoelectron energy and the overall structure of the photoelectron spectrum are much less sensitive to the lack of very high angular momenta. We therefore believe that our angle-integrated PES is converged to within the required accuracy. We further believe that the main features of the angle-resolved spectrum are qualitatively correct.

3. Our absorbing boundary is placed slightly closer to the origin (417 Bohr away) than would be required to fully contain

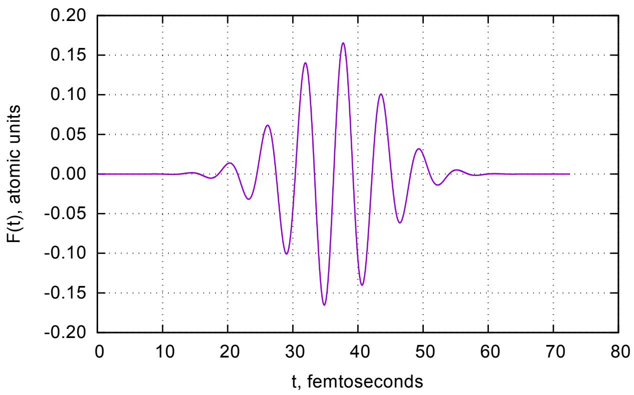

Figure 1. Electric field of the laser pulse used for the calculations.

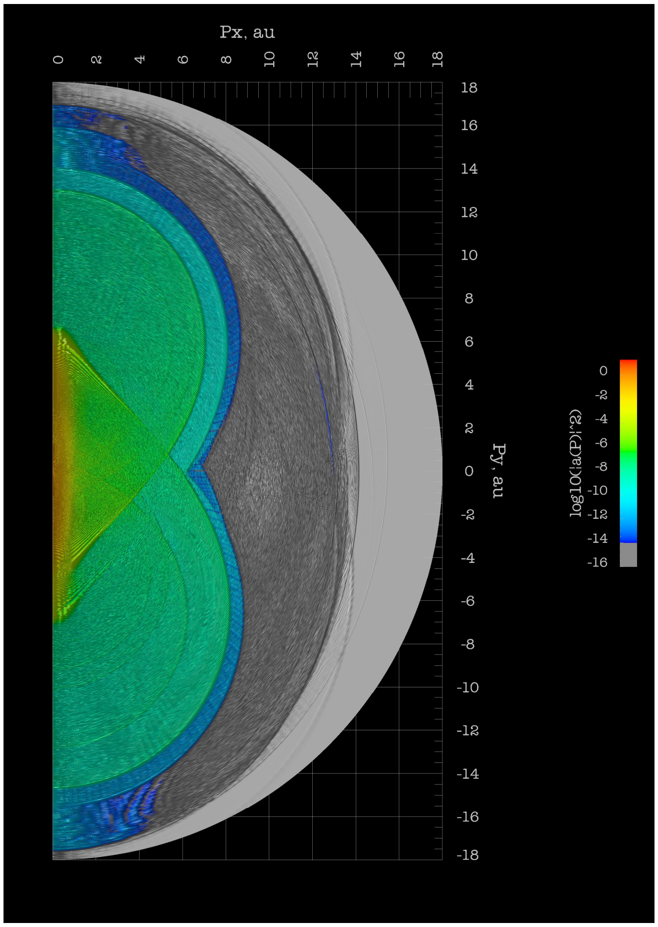

Figure 2. Calculated photoelectron spectrum. See text for details.

all field-driven recolliding trajectories at the nominal peak intensity of the field (the free-oscillation radius at 1×10^{15} W/cm^2 and 1.8 μm is ≈ 260 Bohr). From the visual inspection of the TDSE wavefunction evolution, ionization is essentially complete well before the nominal peak intensity is reached. We therefore do not expect this choice to affect our results, although we can not exclude a slight underestimation of photoelectron yield beyond ≈ 8 Up (2.4 keV).

The calculated photoelectron spectrum is shown in Fig. 2. The vertical (Y) axis gives the final photoelectron momentum (atomic units) along the laser polarization. The horizontal (X) axis shows the final photoelectron momentum perpendicular to the laser polarization. The spectrum is axially symmetric around the Y axis, so that only the right half-plane of the $P_z = 0$ slice of the spectrum is shown. The color code corresponds to the decimal logarithm of the calculated photoionization probability in [jiffy^3/Bohr^3].

The red/orange central part of the spectrum ($P_y \leq 6$, $P_x \leq 1$) represents the direct electrons. The clearly visible vertical asymmetry in the direct- electron distribution arises due to population depletion between subsequent half-cycles of the field. The broader half-cycle green and teal features, centered around $P_y = \pm 6$, are due to the recollision electrons. At least three recollision events are clearly visible: one in the lower hemisphere (green, $max(P_x) \approx 8$) and two in the upper hemisphere (green, $max(P_x) \approx 7$; and teal, $max(P_x) \approx 8$). The three recollision events are consistent with three main ionization bursts before the ground-state population is depleted. Faint signatures of later, slightly more energetic recollision events (blue) are also visible; however, these events do not appreciably contribute to the overall photoelectron yield.

References

1. Morales, F., Bredtmann, T. & Patchkovskii, S. isurf: A family of infinite-time surface flux methods. *arXiv* **1606**, 04566 (2016).
2. Muller, H. G. Numerical simulation of high-order above-threshold-ionization enhancement in argon. *Phys. Rev. A* **60**, 1341–1350 (1999).
